# Supplementary material for: Sustaining Our Relationship: Dyadic Interactions Supported by Technology for People with Dementia and Their Informal Caregivers
Source: Int J Environ Res Public Health. 2022 Sep 2;19(17):10956. doi: 10.3390/ijerph191710956 (PMC9518490; doi:10.3390/ijerph191710956)
Supplement: Supplementary file 1 [file ijerph-19-10956-s001.zip › ijerph-1816981-supplementary File S1.pdf]

I-CARE Semi-Structured Interview Guide – **Post-Intervention**

Usability excerpt

Usefulness

1. Could you tell a little about how you experienced doing the activities together, in general?
2. Did I-CARE help finding things to talk about together?
3. All in all, did you experience the I-CARE sessions as engaging?
4. All in all, did you experience the I-CARE sessions as meaningful?
5. All in all, could you imagine using social technology like I-CARE in everyday life?
6. When thinking of your expectations before using I-CARE, did they match your experiences when actually using the system?
7. Considering the (current) COVID-19 pandemic, how was it to have I-CARE as an alternative activity to do together at home?

User-friendliness

1. How many times per week (on average) did you sit down together to use I-CARE?
2. How long would you say the I-CARE sessions lasted, on average, each time?
3. Which activities did you find most appealing and/or engaging?
4. Which activities did you find the least appealing/ engaging?
5. Did you experience any technical issues while using I-CARE?
